# Supplementary material for: Responses of Soil Microbiota to Different Control Methods of the Spartina alterniflora in the Yellow River Delta
Source: Microorganisms. 2022 May 30;10(6):1122. doi: 10.3390/microorganisms10061122 (PMC9230759; doi:10.3390/microorganisms10061122)
Supplement: Supplementary file 1 [file microorganisms-10-01122-s001.zip › microorganisms-1731885-supplementary.pdf]

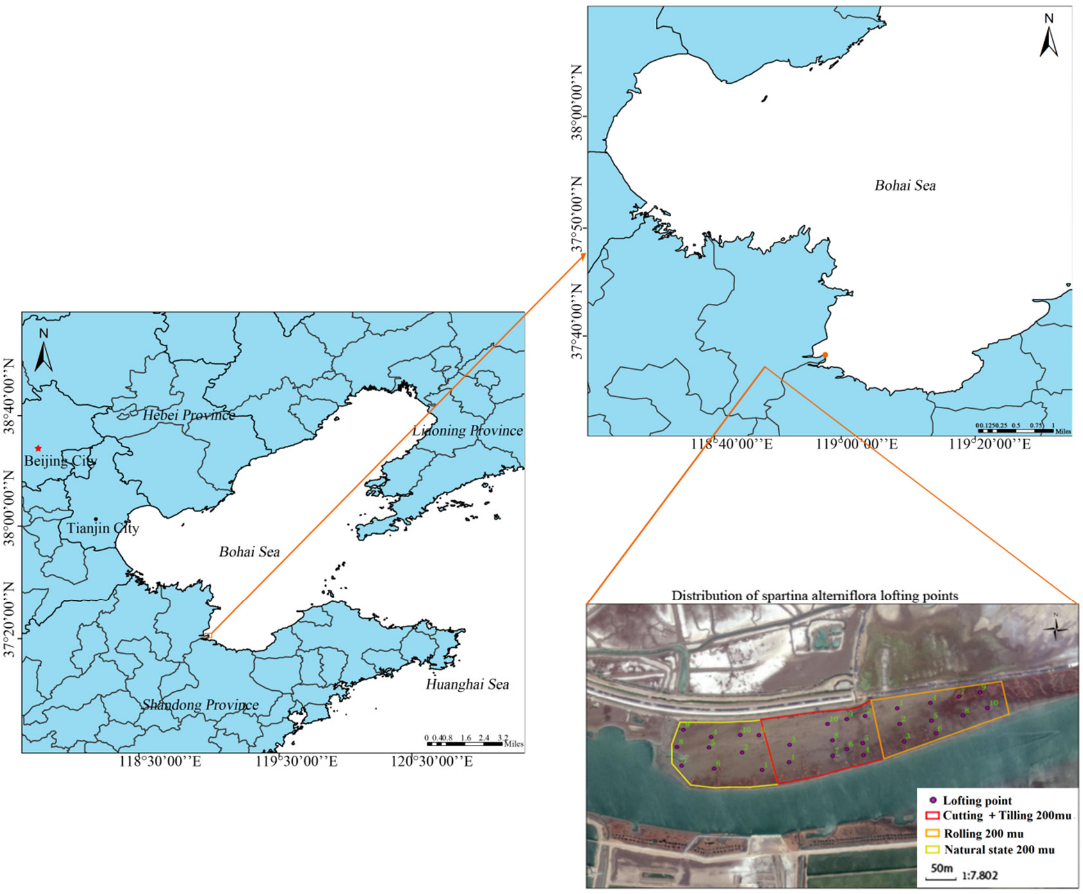

Figure S1. Location of the sampling sites.

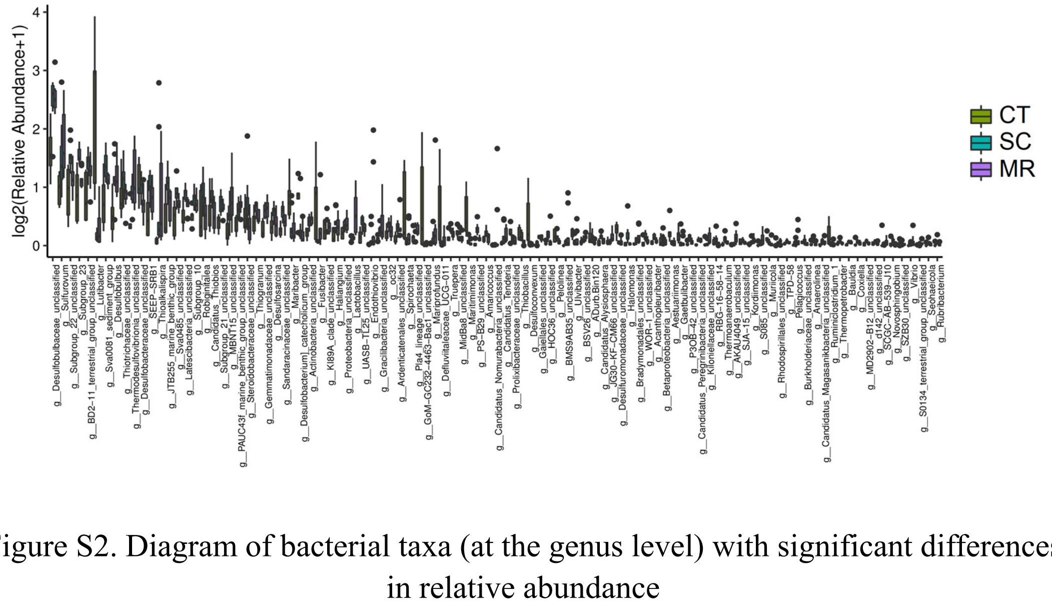

Figure S2. Diagram of bacterial taxa (at the genus level) with significant differences in relative abundance
